# Electrospun nanofibrous membrane for biomedical application

**DOI:** 10.1007/s42452-022-05056-2

**Published:** 2022-05-13

**Authors:** Bomin Yan, Yiwen Zhang, Zhixiang Li, Pinghui Zhou, Yingji Mao

**Affiliations:** 1 School of Life Sciences, Bengbu Medical College, Bengbu, 233030 China; 2 Department of Plastic Surgery, The First Affiliated Hospital of Bengbu Medical College, Bengbu, 233004 China; 3 Department of Orthopedics, First Affiliated Hospital, Bengbu Medical College, Bengbu, 233004 China; 4 Anhui Province Key Laboratory of Tissue Transplantation, Bengbu Medical College, Bengbu, 233030 China

**Keywords:** Electrospinning, Electrospun nanofiber membrane, Biomedical application, Tissue engineering

## Abstract

Electrospinning is a simple, cost-effective, flexible, and feasible continuous micro-nano polymer fiber preparation technology that has attracted extensive scientific and industrial interest over the past few decades, owing to its versatility and ability to manufacture highly tunable nanofiber networks. Nanofiber membrane materials prepared using electrospinning have excellent properties suitable for biomedical applications, such as a high specific surface area, strong plasticity, and the ability to manipulate their nanofiber components to obtain the desired properties and functions. With the increasing popularity of nanomaterials in this century, electrospun nanofiber membranes are gradually becoming widely used in various medical fields. Here, the research progress of electrospun nanofiber membrane materials is reviewed, including the basic electrospinning process and the development of the materials as well as their biomedical applications. The main purpose of this review is to discuss the latest research progress on electrospun nanofiber membrane materials and the various new electrospinning technologies that have emerged in recent years for various applications in the medical field. The application of electrospun nanofiber membrane materials in recent years in tissue engineering, wound dressing, cancer diagnosis and treatment, medical protective equipment, and other fields is the main topic of discussion in this review. Finally, the development of electrospun nanofiber membrane materials in the biomedical field is systematically summarized and prospects are discussed. In general, electrospinning has profound prospects in biomedical applications, as it is a practical and flexible technology used for the fabrication of microfibers and nanofibers.

## Article highlights

- This review summarizes recent research on the application of electrospun nanofiber membranes as tissue engineering materials for the cardiovascular system, motor system, nervous system, and other clinical aspects.
- Research on the application of electrospun nanofiber membrane materials as protective products is discussed in the context of the current epidemic situation.
- Examples and analyses of recent popular applications in tissue engineering, wound dressing, protective products, and cancer sensors are presented.

## Introduction

In the past few decades, nanotechnology has been widely studied and has progressed significantly. Nanomaterials obtained by nanotechnology have received a lot of attention owing to their excellent performance and have had a wide and profound impact on various industries, especially the medical industry.

Electrospinning technology, as a simple and low-cost method to prepare continuous micro-and nano-polymer fibers, has gradually become the focus of research. The electrospinning process utilizes thousands to tens of thousands of volts of high-voltage electrostatic repulsion to produce fibers from a polymer solution and synthesize sophisticated three-dimensional structures [1]. At present, more than 200 kinds of natural polymers and composites, such as gelatin, silk fibroin, chitosan, and collagen, as well as a large number of synthetic polymers such as polycaprolactone (PCL), poly-lactic acid, and poly(lactic-co-glycolic acid) have been applied in electrospinning technology systems [2].

The method of using electrostatic force to produce fibers has been known for more than 100 years [3]. In 1902, Morton and Cooley filed two patents on electrospinning and described a prototype of an electrospinning device [4]. In 1934, Formhals invented and patented an experimental device for the preparation of polymer fibers by electrostatic force. His patent published how a polymer solution formed a jet between the electrodes; this was the first patent to describe in detail a device for preparing fibers by high voltage electrostatic electricity and is considered to be the beginning of the preparation of fibers using electrospinning technology [5]. Since the 1990s, after Reneker et al. demonstrated the feasibility of producing electrospun nanofibers from several polymers, the number of publications on electrospinning has grown exponentially [4].

Electrospinning has been widely applied in the biomedical field. On the one hand, it benefits from the rapid development of nanotechnology, and on the other hand, it benefits from its unique material properties. The nanofibers obtained by electrospinning have strong plasticity, a flexible structure, and a large surface area-volume ratio, which can enhance cell adhesion, proliferation, and differentiation activities. Recent literature has shown that electrospinning techniques allow the nanosurface modification of tissue engineering materials to control protein adsorption and biochemical construction of protein layers, thus building “bottom-up” nanoscale features that can be used to guide surface hydrophilicity, oxide layer thickness, or functional group distribution to form extracellular matrix (ECM)-like nanostructures that mimic biological environments and influence cell behavior, signal transduction, and nutrient transport [6]. Therefore, their application in the field of tissue engineering has been widely noticed and studied. In addition, electrostatically-spun nanofiber membrane materials have shown increasing applicability to the construction of drug delivery systems, not only due to their good biocompatibility as well as safety, but also due to their large specific surface area-volume ratio, which allows drugs to maintain a high effective surface area at a lower cost [7]. More notably, electrostatically-spun nanofiber membranes also provide flexibility in the choice of drug-carrying materials and offer a variety of methods to carry drugs, such as coating and encapsulation [8]. It is worth mentioning that at a time when the Coronavirus epidemic continues to make significant impacts on people’s lives, electrospinning nanofiber membranes have the advantages of high porosity, pore size adjustability, and a small fiber diameter, providing grounds for many new ideas regarding the production of medical protective gear [9]. Recently, an increasing number of biomedical products made of electrospun nanofibers have been approved for clinical use [10].

In recent years, the electrospinning technology boom has led to the application of electrospinning equipment from the laboratory to the market. Several manufacturers have developed new methods to improve the production capacity of electrospinning products by adapting traditional electrospinning, and have succeeded in making electrospinning nanofiber products available in a relatively safe way with relatively simple operation [11]. In the medical field, several electrospun nanofiber membrane materials have been successfully patented, commercially produced, and used for surgical implants, dressings, and medical devices [12].

In this review, the basic principle of electrospun nanofiber membrane preparation is briefly introduced, and the applications of electrospun nanofiber membranes in various medical fields in recent years are classified and discussed. In the following section, we introduce the basic principle of electrospinning technology and its improvement and development in recent years. In Sects. 3–6, we respectively introduce, in detail, the application of electrospun nanofiber membranes in stent engineering, wound dressing, tumor diagnosis and treatment sensor development, protective equipment development, and other medical fields in recent years. In the last section, we summarize the wide application of electrospun nanofiber membranes in the medical field more broadly and discuss the development prospects of electrospinning technology.

## Fundamental principles of electrospinning

### Basic electrospinning equipment and workflow

**Fig. 1:**
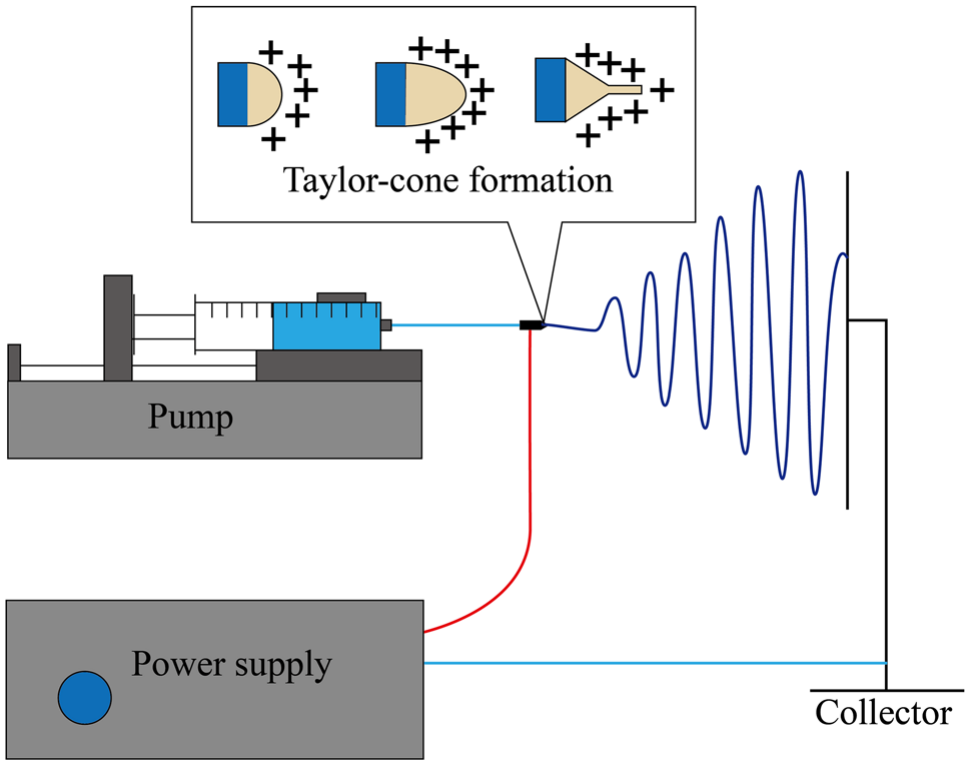
Schematic diagram of typical electrospinning device [14]

The most basic equipment used in electrospinning is a high voltage power supply, a flow controller, a spinneret (i.e., a syringe pump with a needle), and a grounded collector [13, 14] (Fig. 1). The polymer solution/melt used for electrospinning is extruded from the spinneret at a certain speed under the control and propulsion of the syringe pump. Due to surface tension, the initially extruded liquid takes the shape of suspended spherical droplets. When a high voltage power supply is connected, a high voltage is applied between the spinnerette and the grounded collector [15]. The electrostatic repulsion of the high voltage stretches the droplet on the tip of the needle from its normal spherical shape to a conical shape (also known as a Taylor cone). When the voltage exceeds a given threshold, the electric field force will overcome the surface tension of the droplet and cause the filament to extend from the tip of the cone, creating one or more jets of charged polymer solution [16]. During the charged jet jetting process, factors such as the solvent volatilization of the polymer solution (solution electrospinning) or solidification of the polymer melt (melt electrospinning), as well as the effect on the charged jet under the influence of electric field forces will cause unstable stretching and whipping [17, 18]. Eventually, the charged jet rapidly refines and solidifies during this motion, and the resulting solid fibers are attracted, deposited, and collected by an electrically charged grounded collector. In this manner, polymer fibers with nano to submicron fibers can be obtained [19].

Continuous nanofibers with good morphological characteristics cannot be prepared using traditional methods such as drawing, self-assembly, phase separation, and template synthesis [20]. Electrospinning technology is widely used because it can work synergistically through multiple mechanisms to produce nanofibers with continuous, highly ordered, and smooth morphology, and different factors can be regulated to circumvent adverse effects [21]. Compared with other methods, electrospinning is simpler, more versatile, and less costly. In addition, electrospun nanofibers have the advantages of a small diameter, large surface area, high aspect ratio, and high flexibility [22].

The electrospinning process is influenced by many adjustable variables, the three most important of which are the spinning conditions, the polymer solution, and the environmental conditions [18]. The spinning conditions mainly include the intensity of the applied voltage, the flow rate controlled by the syringe pump, and the tip-to-collector distance; the influence of the polymer solution on electrospinning usually comes from its concentration, conductivity, viscosity, surface tension, and polymer molecular weight; and the environmental influences are mainly ambient temperature and humidity [14]. By controlling the materials and methods of electrospinning, the composition, structure, and properties of nanofibers can be designed for specific applications.

### Drug loading strategies for electrosspun materials

In recent years, in addition to loading drugs directly onto the surface of flat nanofiber membranes, most researchers have chosen to use drug-hybrid electrospinning scaffolds such as the recently popular coaxial electrospinning technique, co-blended electrospinning technique, and emulsion electrospinning technique to produce drug-loaded nanofiber membranes to achieve the controlled release of drugs in electrospun nanofiber membranes over long periods [23]. Coaxial electrospinning is the simultaneous electrospinning of two solutions through two coaxially fed capillary channels in the same needle to produce polymeric micro-nanofibers with different internal and external parts of the core-sheath structure, while being able to encapsulate the drug into the core-sheath structure. It is considered an effective strategy to achieve slow drug release [24]. Emulsion electrospinning and co-blended electrospinning are also used to fabricate core-sheath structures capable of achieving sustained drug release. The difference between these two methods is that emulsion electrospinning utilizes a water-in-oil emulsion structure and is mostly used for water-soluble drugs loaded into the core layer, while co-blended electrospinning uses an oil-in-water emulsion structure, which is mainly used to load fat-soluble drugs into the core layer to form a core-sheath structure [24].

### Improvement and development of electrospinning technology in recent years

The production rate of the laboratory-scale equipment needed to needle electrospinning is low, and such equipment is unsuitable for large-scale industrial manufacturing [14]. In recent years, researchers have continued to try to develop new means to expand electrospinning production. Researchers have successively used different methods of design, such as single-needle and multi-needle modification. For example, a single nozzle with a fluted tip can be used to create multiple jets instead of the traditional single needle to increase production. But they are still not satisfactory [11, 25]. The single-needle modification technique does not provide significant capacity improvement, while the multi-needle design suffers from problems such as electrostatic field interaction between needles and needle clogging [11]. Recently, needleless technology has started to gain attention.

In needleless technology, traditional needles are replaced with different new spinnerets and hence the problems of electrostatic field effects between needles and needle clogging are avoided. The spinnerets used in needleless electrospinning can be divided into two main categories: rotary spinnerets and fixed spinnerets [26]. Rotating spinnerets mechanically rotate the polymer solution, while stationary spinnerets typically use different means, such as magnetic fields or bubbles, to assist in the initiation process [27]. Different needleless electrospinning forms of self-guided multi-nozzles, although present in different forms, can improve the production efficiency of electrospun nanofiber membrane materials to a large extent.

Alternating current (AC) electrospinning technology uses alternating current instead of conventional direct current (DC) as the power source. The jet is generated from the droplet and forms a nanofiber plume in the stretching electric field. The fiber plume accelerates with the electron wind generated under the action of the electric field force and slows down beyond a certain distance (3–4 cm). The fiber is then pulled thin and eventually forms a fiber deposit due to solvent evaporation.

From 2004 onwards, when Royal Kessick was the first to publish a study on the use of alternating current for electrospinning [28], it was gradually discovered that AC electrospinning is more advantageous to DC electrospinning in several aspects [29]. On the one hand, the efficiency of AC electrospinning is higher. Multiple jets can be formed simultaneously on the droplet surface under the action of AC, and the yield is more than 20 times that of DC electrospinning under the same experimental conditions [30]. On the other hand, the jets of AC electrospinning are more stable during the stretching process and they do not carry much charge due to the high AC voltage, so there is no need to use the attraction of grounded conductive collectors. The self-bundling of the fiber plume also makes it easy to twist and knot it into yarns to form more stable nanofibers. More importantly, AC electrospinning is relatively more controllable. During this process, the stretching of the fiber plume is mainly affected by the electric wind or corona wind which appears around the metal electrode. The speed of the electric wind or corona wind is proportional to the AC voltage within a certain range, so to a certain extent it can control the formation of nanofiber morphology and properties by controlling the voltage and frequency of AC [31]. However, at present, the application of AC electrospinning also has its relative shortcomings. For example, the fiber diameter that is formed only reaches 300–500 nm. This is relatively bulky compared with DC electrospinning, which can be as fine as 1 nm [32]. Recently, the study of nanofibrous yarn materials obtained via AC electrospinning assembly has been favored in biomedicine. Nanofibrous yarn materials consist of multiple bundles of very fine nanofibers interconnected to form a material structure with high mechanical integrity [33] and are commonly used in the fabrication of various biomedical materials such as sutures [34]. There is no doubt that the development of AC electrospinning technology will attract great attention in the future.

## Application of electrospun nanofiber membranes in tissue engineering

The rapid development and breakthroughs in tissue engineering have contributed significantly to the repair and reconstruction of damaged tissues and organs, especially the repair of bone and cartilage, heart and blood vessels, skin, and other tissues and organs, thereby attracting increasing attention.

Cytokines affecting cell growth and differentiation, suitable cell sources, and suitable scaffolds for tissue cell regeneration are the three basic components of tissue engineering construction [35]. The key to tissue engineering is to select the right biomaterials and construct the right scaffolds. A scaffold material must be biocompatible and biodegradable, promote cell penetration and tissue growth, provide biomechanical support, have a low price, and be easy to obtain, produce, and handle [36]. Many different biomaterials have been extensively tested to meet these requirements; however, electrospinning materials stand out from the rest.

Using electrospinning to produce nanofiber materials is popular in tissue engineering. Compared with other fiber formation processes, such as self-assembly and phase separation, it is a relatively simple and is a more cost-effective method to produce fibrous scaffolds with submicron pore structures and interconnected fiber diameters [37]. The materials that are used for tissue engineering and those that are used as carriers into the human body for organ repair need to be easily absorbed and easily degraded. Electrospinning technology, with its drug-carrying material selection flexibility and versatile drug-carrying methods, can facilitate researchers to solve this challenge well. Additionally, the performance of electrospun nanofiber materials is affected by the fiber size. According to the observation and analysis of the human body by modern biotechnology, the ECM of almost all connective tissues (such as the skin, cartilage, and bones) in the human body is similar in structure to nanofibers. Through electrospinning technology, it is not only possible to control the structure and morphology of the produced nanofiber membrane material through parameters such as textile conditions, it is also possible to simulate the ECM structure, including the porosity, mechanical properties, and area-to-volume ratio. Modification of the nano surface of the tissue engineering material can also be achieved using electrospinning technology, thus allowing the construction of the surface protein layer and the distribution of functional groups and surface hydrophilicity to be controlled. In addition, it can provide an environment similar to the original structure of natural tissue ECM, thus ensuring—as much as possible—the integrity of cellular nutrient transport and permeability, and greatly improving the biocompatibility of the obtained tissue-engineered scaffolds [38, 39].

### Application of electrospun nanofiber membranes in cardiovascular tissue engineering

Cardiovascular disease (CVD) has long been the leading cause of death in the world, posing a great threat to human health. CVD includes coronary artery disease (such as angina pectoris and myocardial infarction), stroke, valvular heart disease, myocarditis, cardiomyopathy, aortic aneurysm, artery disease, thromboembolic disease, and venous thrombosis [40]. All cardiovascular diseases have their own unique and hidden pathogenesis. For many years, the main clinical use of surgery and cell therapy was to treat cardiovascular system diseases [41]. However, conditions such as the high risk of rejection and unnecessary structural changes in the body condition place many limitations on the use of traditional therapies in various ways. The cardiac muscle is a kind of electroactive tissue, and the regeneration potential of cardiomyocytes is very small, which leads to their weak self-repairing ability; as such, repairing cardiomyocytes remains a problem. However, decades after the introduction of cardiovascular tissue engineering, it has evolved to induce the creation of functional heart tissue, providing a great boost to the treatment of CVD [42]. Electrospun nanomaterials are good as conductive and inducer scaffolds and are suitable platforms for heart and blood vessel cells, thus providing a new approach for cardiovascular tissue engineering. There has been extensive research on vascular grafts, artificial heart valves, and myocardial tissue engineering in particular in recent years.

#### Application of electrospun nanofiber membranes in vascular grafts

Although the gold standard for vascular replacement is still autografting, the use of autologous grafts is limited by donor shortage and secondary site injury, thereby making autografting difficult to deal with for all types of vascular injury. Artificial vascular transplantation has become a research hotspot in the field of vascular replacement. Although large vessel grafts have been successfully used in the clinic, the use of small-diameter vessels (diameter < 6 mm) remains a significant challenge, as most existing vascular graft materials are prone to secondary intimal hyperplasia and thrombosis [43]. Therefore, there is an urgent need for new strategies to develop novel small-diameter grafts with rapid endothelialization and without thrombotic complications [40].

**Fig. 2:**
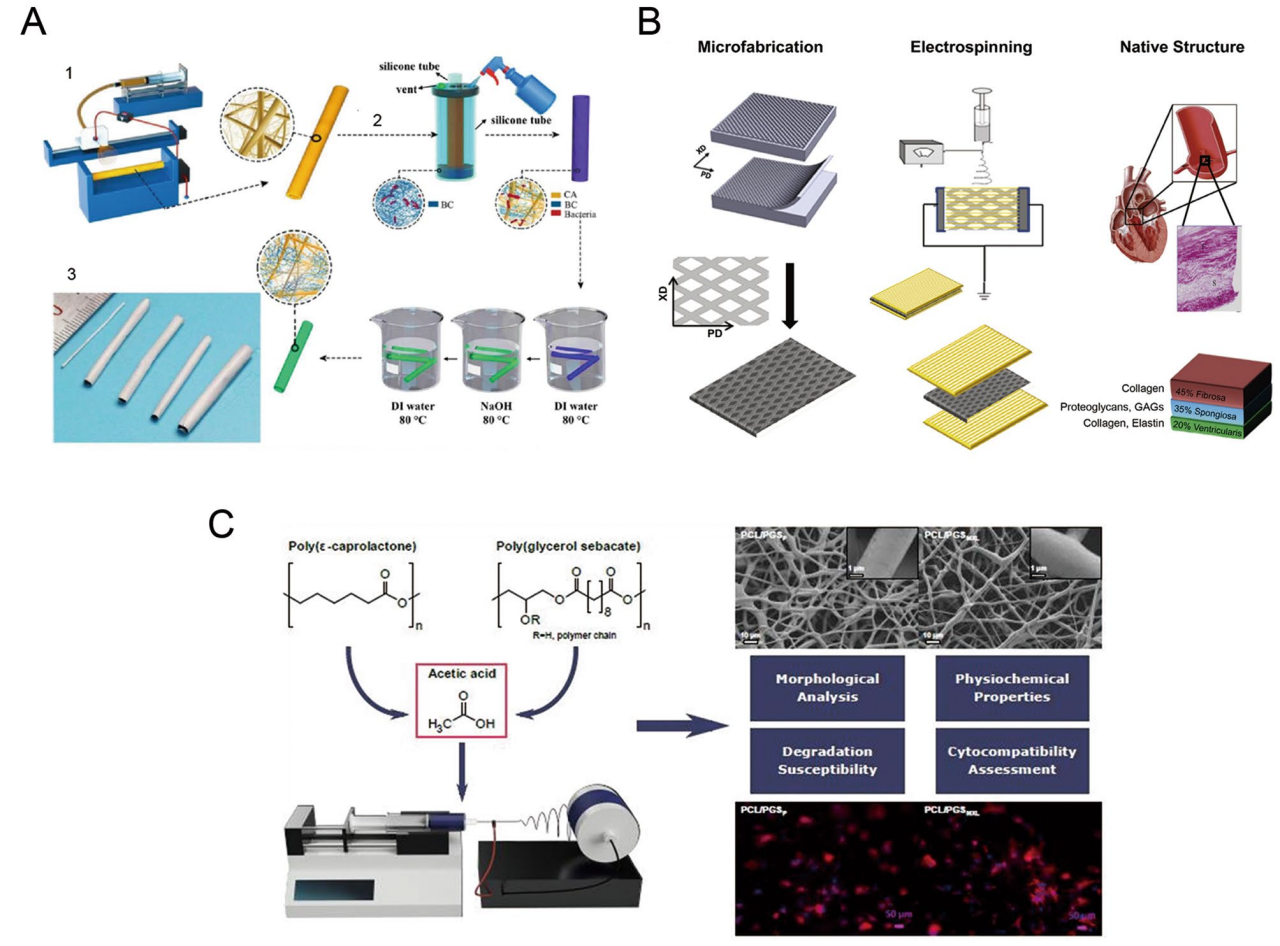
**a** Small diameter composite vascular grafts combined with nanofilament bacterial cellulose (BC) and submicrofiber cellulose acetate (CA) were prepared by electrospinning and step-by-step in situ biosynthesis [45]. Reprint with permission from The Royal Society of Chemistry; **b** Manufacture the microprocessed polyglyceride sebacate (PGS) sheet; The PGS sheet was placed between two aluminum electrodes and connected to the ground. PGS/Polycaprolactone (PCL) fibers were placed on both sides of the PGS layer for electrospinning. The use of this fibrous membrane stent in valve repair [52]. Reprint with permission from Elsevier; **c** PCL and PGS fibers have been used in cardiac tissue engineering [57]. Reprint with permission from Elsevier

To solve the aforementioned problem, many researchers have focused on electrospun nanofiber membrane materials. For example, Kuang et al. used a simple electrospinning technique to fill nanofibers with salvianolic acid B and heparin extracted from the traditional Chinese herbal plant *Salvia miltiorrhiza* to construct the vascular lining. This method was able to effectively prevent acute thrombosis and promote the rapid endothelialization of blood vessels [44]. Wan et al. combined electrospinning and progressive in situ biosynthesis and prepared a small-diameter composite vascular graft combined with nanofilament bacterial cellulose and sub-microfiber cellulose acetate, which was also effective in reducing the thrombotic potential of the graft and enhancing endothelialization [45] (Fig. 2a). Jiayin et al. electrostatically spun different thicknesses of PCL onto porous polyglycerol sebacate (PGS) and found that thicker PCL fibers which also have higher porosity were more capable of promoting angiogenesis and showed better mechanical properties than thinner groups, thus demonstrating the importance of porosity and fiber diameter in the design of vascular grafts [46]. Kang et al. utilized a co-blended electrospinning technique to create a combination of natural ECM and synthetic degradable polymers, and then used them as vascular grafts for animal experiments. The grafts exhibited sustained hyaluronic acid release and promoted the regeneration of smooth vascular muscle [47]. Similar strategies have emerged in recent years, and it can be predicted that these biomimetic vascular grafts using electrospun nanofibers will play an important role in vascular reconstruction and regeneration.

#### Application of electrospun nanofiber membranes in artificial heart valves

Valvular heart disease affects approximately 2.5% of the global population. According to a new report [48], as life expectancy increases in industrialized countries, the incidence of valvular heart disease will triple after 2050 owing to age-related valvular degeneration [49]. At present, the main treatment methods include valvular repair and replacement. The former is the first choice for young patients with aortic regurgitation. Valvular replacement remains the treatment of choice for patients with severe valvular dysfunction, with more than 300,000 patients globally reported to undergo valvular replacement surgery annually [50].

At present, most valves used in clinical practice are made from animal pericardial tissue. These valves have complex post-processing procedures; the complexity of their ingredients and batch instability/variability make quality control difficult and they come with a high risk of viral infection and costs that the average person cannot afford [49]. Therefore, finding a new valve source to replace the traditional domesticated animal sources has become the new research focus [51]. Pericardial leaflets are more prone to calcification, injury, and deterioration; thus, good hemodynamic performance and anti-calcification performance are required for new valve materials.

The electrostatically spun nanofiber membrane material is easy and inexpensive to obtain, and it can well avoid the risk of infection that may occur using a traditional valve extracted from an animals. This provides a new avenue for developing suitable valve materials in clinical practice. Nafiseh et al. developed elastic scaffolds with tunable anisotropic mechanical properties by assembling microfabricated PGS and fibrous PGS/PCL electrospun sheets. They report that these materials can effectively support the growth of valvular mesenchymal cells and mesenchymal stem cells in three-dimensional structures and promote the deposition of heart valve ECM [52] (Fig. 2b). Using the electrospinning technique, Jana et al. prepared a nanofibrous material that can simulate a three-layer natural valve. The experimental results showed that this material has good morphological, mechanical, and biological characteristics, which can be used as a basis for the development of a tissue-engineered heart valve structure for the treatment of heart valve disease [53]. Poly (L-lactic acid-co-ε-caprolactone) (PLCL) and silk fibroin (SF) were blended in different proportions to fabricate electrospun scaffolds. The results showed that the electrospun scaffolds with a PLCL/SF ratio of 80/20 had good mechanical properties, anti-calcification ability, and good cellular compatibility, thereby having great potential as a tissue engineering material for heart valves [51]. Therefore, more tissue-engineered heart valves constructed by electrospun nanofiber membrane materials are being researched, offering the potential for suitable artificial valve materials.

#### Application of electrospun nanofiber membranes in myocardial tissue engineering

Myocardial infarction (MI) is one of the leading causes of death worldwide. It is a structural and functional disorder caused by the sudden obstruction of one or more coronary arteries, which leads to changes such as reduced cardiac contractile power and finally to chronic or congestive heart failure. Studies have shown that only 1% of the cells in the heart muscle tissue itself can regenerate each year and this number drops to 0.3% at age 20 and to zero by age 75 [54]; therefore, there is high demand for alternative therapeutic strategies and approaches to repair damaged post-MI tissue, a common strategy known as cardiac tissue engineering (CTE). CTE has become a research hotspot in the field of tissue engineering owing to its great potential for clinical applications.

Electrospun nanofiber membranes can produce continuous micro-nano polymer fibers, which have a biomimetic structure that can simulate the ECM structure of cardiomyocytes well [55]. Richard et al. used electrospinning to design a non-invasive PCL/gelatin scaffold to obtain a nanofiber woven mesh that enhances the recruitment of macrophages, the expression of pro-angiogenic cytokines, vascular endothelial growth factor, the placental growth factor, and the corresponding host angiogenesis mechanisms [56]. In addition, the homogenous defect-free felts of PCL and PGS have been successfully electrospun in the acetic acid solvent. The results showed that acetic acid had no negative effect on the ability to form a uniform fiber with a diameter of 1.3 μm without defects [57]. Moreover, the mechanical properties of electrospun nanofiber membranes are higher than those of human myocardial tissue, which may be beneficial to reduce infarct dilation and left ventricular remodeling [57] (Fig. 2c). It is not difficult to imagine that the rapid development of cardiac tissue engineering will provide more options and strategies for the treatment of ischemic myocardial injury and infarction.

### Application of electrospun nanofiber membranes in bone and cartilage

Bone and cartilage tissue is one of the most vulnerable organs in the human body, which has more than 200 bones of different shapes, sizes, and functions. The loss of bone function due to diseases such as fractures and osteoporosis will inevitably lead to a loss of life quality. Fortunately, bones exhibit a unique ability to regenerate can be healed without structural or functional damage. However, whenever the size of the defect exceeds the healing capacity of the osteogenic tissue, the site does not regenerate completely and the problem needs to be addressed using a bone graft [36]. Traditional solutions involving bone grafts are limited by their high cost and lack of tissue donors.

In recent years, people have tried a variety of approaches to overcome such large bone defects, by combining bone tissue engineering with biomimetic scaffolds. According to the research progress of recent decades, osteoinduction and inorganic biomimetic scaffold materials are continuously optimized, and scaffold structures similar to the microstructural design of original bone tissue are continuously updated [58].

The application of nanofibrous scaffolds prepared by electrospinning in bone tissue engineering has attracted a lot of attention since it came into public view. The potential of electrospun nanofibrous materials as bone substitutes is promising because electrospinning can simulate ideally the micro-nano morphology of the fiber structure similar to that of natural bone ECM [59].

#### Application of electrospun nanofiber membranes in bone tissue engineering

Bone defects, often caused by trauma, tumors, osteomyelitis, abnormalities, and degenerative diseases, have a significant impact on patient quality of life. The repair of large bone defects remains a clinically difficult problem because of the limited supply of traditional bone graft tissue and the increasing clinical demand. Therefore, according to the current situation, it is particularly important to develop bionic tissue engineering scaffolds for bone regeneration [60]. In tissue engineering, scaffolds provide a platform for cell attachment, proliferation, and differentiation, and play an important role in regulating cell behavior through a unique microenvironment [61].

**Fig. 3:**
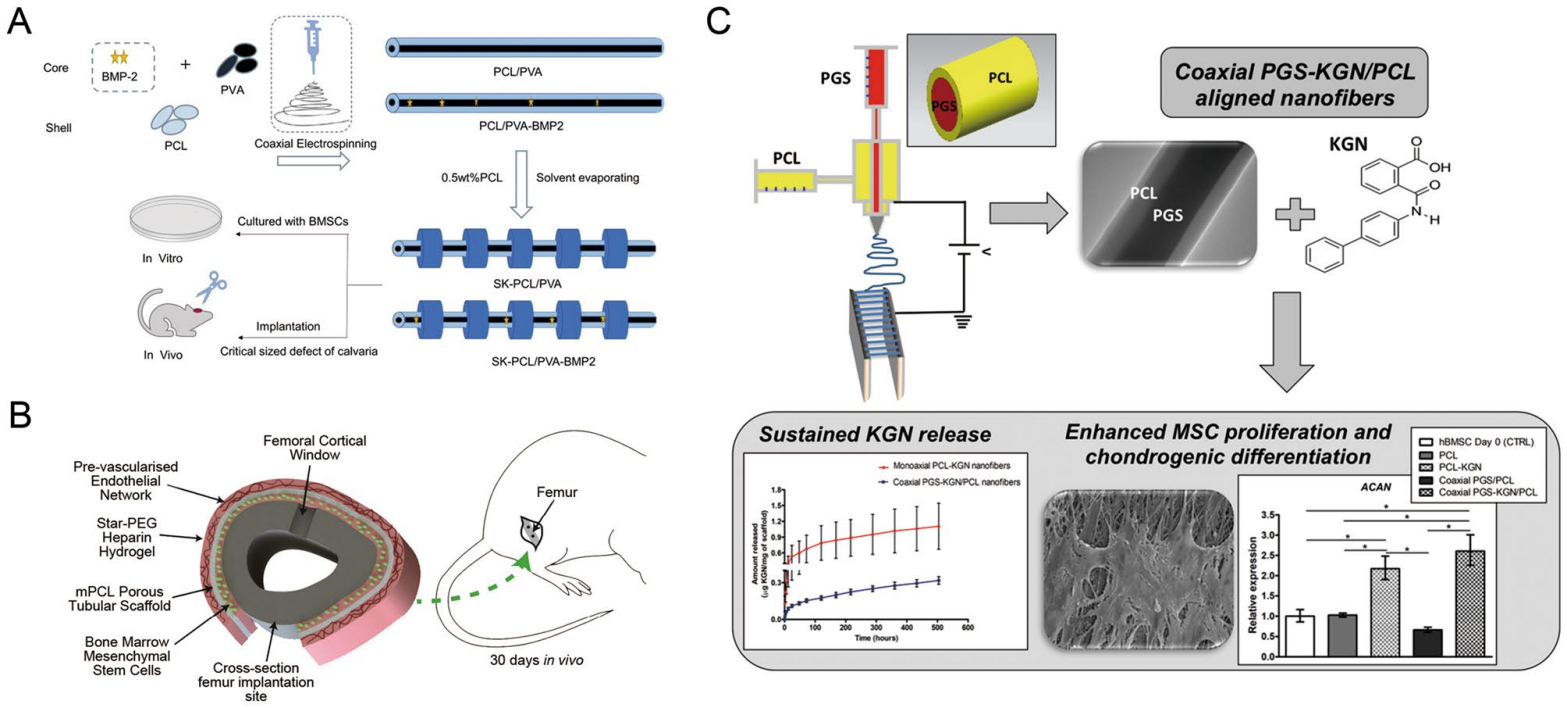
**a** Preparation and application of shish-kebab (SK) structure electrospinning scaffold for repairing skull defect in vivo [62]. Reprint with the permission from American Chemical Society; **b** Tissue engineered periosteum is used to repair bone defects [68]. Reprint with permission from Elsevier; **c** Fabrication of coaxial PGS-KGN/PCL aligned nanofibers and application of the fiber membrane in cartilage repair [73]. Reprint with permission from Elsevier

Ideal properties for bone repair applications include mechanical strength, immediate limb stability, biological activity, interconnecting pores (blood supply, gas exchange, bone growth, and vascular penetration), degradability, controlled delivery of bioactive molecules/drugs, bone fusion, osteoinduction, bone conduction, and prevention of biofilm infection [62] (Fig. 3a).

In recent years, nanofibrous materials are an attractive option for bone tissue regeneration applications because of their 3D nanostructures that mimic the natural ECM; they are excellent in vitro and in vivo compatibility [63–65]. Electrospun nanofibers have attracted extensive attention because of their high drug loading efficiency, convenient component control, and morphology design [60].

The use of electrospun nanofiber materials for the repair of different types of bone defects has become a research hotspot in recent years. For example, Perumal et al. studied the ability to repair critical-sized femoral bone defects in New Zealand white rabbits using a double-layer nanocomposite coating of polycaprolactone and nano-hydroxyapatite impregnation and electrospinning. The results showed that the nanocomposite-coated implants had controlled in vivo degradation and improved bioactivity, which demonstrated that these coated implants could be used as a bioabsorbable implant material for the repair of critical segmental bone defects [66]. Venugopal et al. electrospun PCL nanofibers with hexadecanoic acid, octadecanoic acid, N, N-diisopropylamine, and phytochemicals isolated from the medicinal plant *Wattakaka volubilis*; the results showed that PCL nanofibers mixed with this phytochemical substance could significantly promote the growth and proliferation of primary human meniscus and osteoblast-like cells [67]. In addition, some studies have used fractional nanostructured bone carrier MP2 core–shell nanofibers to repair cranial defects of critical size in rats [62]. The use of electrospun tissue engineering materials, including all kinds of long bone and flat bone, has been proposed extensively.

The periosteum plays an important role in bone development and injury healing. However, there are few studies on artificial periosteum, which are also limited by the complexity of their construction and the biological risk of clinical application. Nevertheless, there is an accumulating body of research on the repair of bone defects by periosteum regeneration; the classic tissue-engineered periosteum used for bone defects in rats is shown in the figure [68] (Fig. 3b). For example, it has been reported that icariin (ICA) was introduced into poly(ε-caprolactone) (PCL)/gelatin nanofibers by coaxial electrospinning technology to prepare an artificial periosteum. The results of this study demonstrate that the ICA-loaded PCL/gelatin electrospun membrane has great potential to promote bone regeneration as a bionic artificial periosteum [69]. In some studies, vascular endothelial growth factor (VEGF) was encapsulated with hyaluronic acid-polylactic acid core–shell structure by collagen self-assembly and microsol electrospinning techniques. Complete regeneration of periosteum and bone tissue is achieved by inducing endogenous cambium in vivo [70]. These studies indicate that bionic periosteum has proved to be effective and multifunctional in triggering periosteum and bone regeneration and provides a promising strategy for the clinical repair of bone defects.

#### Application of electrospun nanofiber membranes in cartilage tissue engineering

Articular cartilage is a very durable tissue that helps the body withstand significant compression and shear forces. However, after a traumatic injury, due to the lack of blood vessels in the natural cartilage, the potential for cartilage to regenerate internally is weak. If left untreated, focal cartilage damage can lead to degenerative osteoarthritis [71]. In clinical practice, autologous transplantation of chondrocytes is considered an ideal strategy. However, it is difficult to extract a sufficient amount of cartilage during natural cartilage grafting, and this method is prone to morbidity in its donor area [72]. Loading stem cells with the ability to differentiate cartilage into tissue-engineered scaffolds is gradually becoming a popular research direction for cartilage tissue repair. The structure of electrospun nanofiber membranes not only makes them highly valuable as osteogenic scaffolds, it has also led to their gradual exploration as chondrogenic scaffolds to repair cartilage defects. This enables the fabrication of fibrous scaffolds with high porosity and large surface areas that mimic the nanoscale and arrangement of collagen fibers in the natural extracellular matrix of articular cartilage [73] (Fig. 3c).

Many recent studies have reported the application of electrospun nanofiber membranes for cartilage repair. For example, Martin et al. developed an electrospun cell-free fibrous hyaluronic acid scaffold that provides a specially designed factor to enhance cartilage repair: stromal cell-derived factor-1α (SDF-1α) and transforming growth factor-β3 (TGF-β3). Experiments showed that the SDF-releasing scaffold produced a lower cartilage healing response, which should guide the search for alternative growth factor combinations [74]. Begum et al. employed an electrospinning technique that uses a chondro-induced cellulose and silk polymer blend (75:25 ratio) to simulate the in vivo nanofiber ECM of cartilage [75]. Girao et al. employed a continuous adaptive electrospinning spraying setup to construct a grading system consisting of PCL fibers and polyethylene glycol sacrificial particles as an excellent biomimetic material for CTE [76]. The use of coaxial PGS-Kartogenin (KGN)/PCL-directed nanofibers to repair cartilage defects has also been proven to be a good strategy [73]. Although there are many challenges in translating cartilage therapy into clinical practice, it is believed that with the advancement of research, electrospun nanofibrous membrane materials will play their role in the cartilage repair field.

### Application of electrospun nanofiber membranes in neural tissue engineering

The nervous system is the most complex system in the human body. It consists of the central nervous system (CNS) and the peripheral nervous system (PNS). Globally, 6.8 million people die each year as a result of traumatic brain and spinal cord injuries, neurodegenerative diseases, and neurological diseases such as stroke [77].

CNS does not regenerate under normal circumstances, and the current medical methods for damaged CNS mainly focus on stabilization and prevention. The treatment for PNS injuries is relatively simple. The nerve fibers of PNS have a remarkable ability to regenerate and almost completely return to normal function after crush injury or Sunderland Type II injury, under the control of Schwann cells through their unique ability to dedifferentiate into cells that drive the healing process [78]. At present, the treatment of PNS injury includes nerve autograft and allograft, but problems include the shortage of the donor’s nerve, disease at the donor site, and difficulty in abnormal regeneration [79]. In recent years, neural tissue engineering and regenerative medicine have provided new strategies for traditional transplantation methods. Nerve tissue engineering uses an external biomaterial scaffold as a platform to allow cells to migrate to the site of injury and repair the tissue [79, 80].

Recent attention has focused on the use of biomaterials as scaffolds for axon growth, loaded with cells and/or neurotrophic or neuroprotective factors [81, 82]. In general, researchers have chosen polymeric materials with excellent electrical conductivity to mimic the natural ECM during neural development. Common conductive polymers, including polyphenylene, polypyrrole (PPY), and polythiophene, have been extensively studied for the fabrication of neural tissue engineering scaffolds [82–84].

**Fig. 4:**
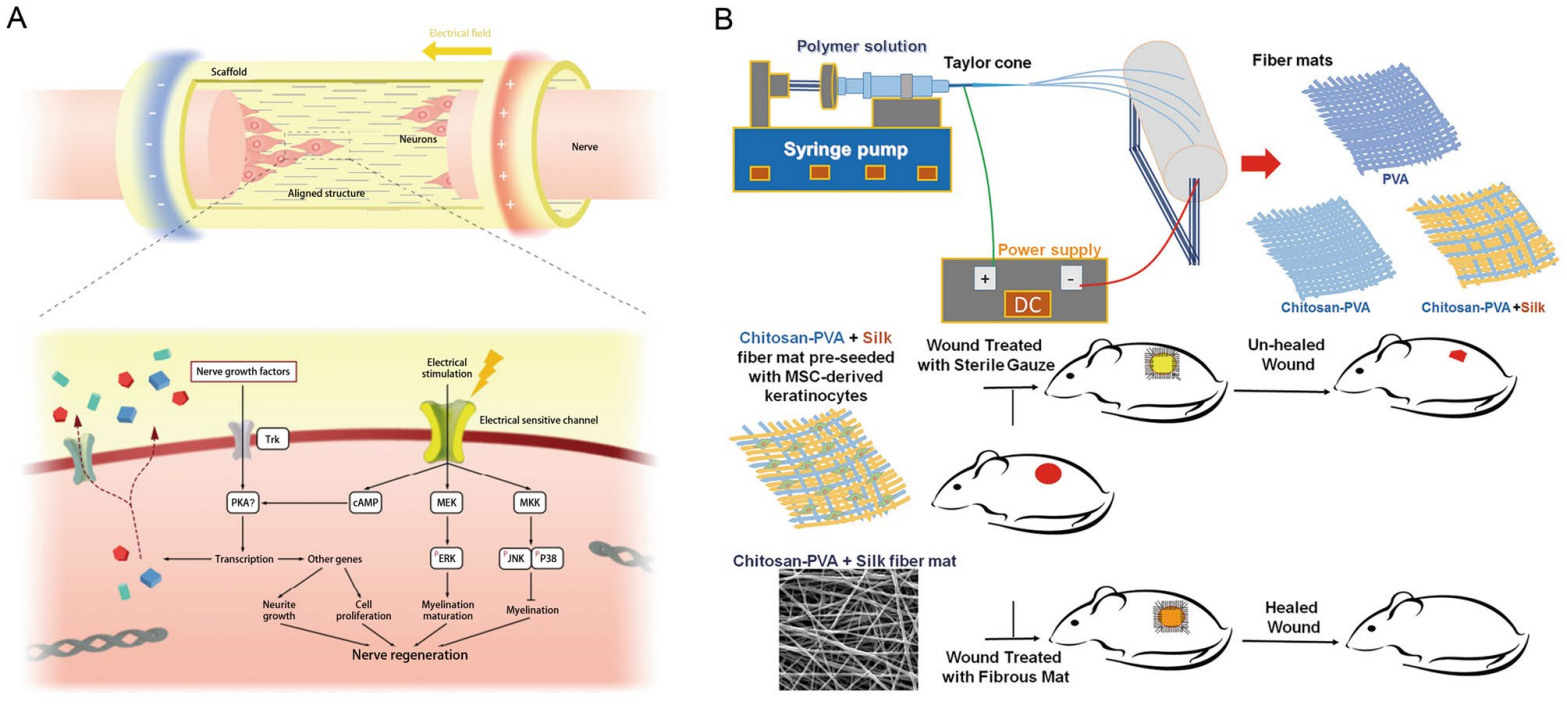
**a** The electrospinning PPY/SF conductive composite nanofiber membrane scaffolds and the possible mechanisms of the promoted neural regeneration [85]. Reprint with permission from Elsevier; **b** Hybrid chitosan (CH), polyvinyl alcohol (PVA), and silk mat were prepared by electrospinning method and applied to the full-thickness wound 6 excision rat model to evaluate the wound healing potential of transplanted CH-PVA + silk mat preimplantation of MSC-derived keratinocytes [89]. Reprint with permission from Springer Nature

Electrospun nanofiber membranes have been widely studied and applied in the treatment of PNS. Zhao et al. prepared PPY/SF conductive composite nanofiber membrane scaffolds using electrospinning and 3D bio-printing. Schwann cells inoculated on these scaffolds were electrically stimulated, and thus showed enhanced viability, proliferation, and migration, as well as upregulated expression of neurotrophic factors. This can effectively promote axon regeneration and myelin sheath regeneration in vivo, and thus promote nerve regeneration and functional recovery [85] (Fig. 4a). Idini et al. showed that the loading of glycosaminoglycan (GAG) onto PCL nanofiber membrane scaffolds can enable GAGs to exert their promoting effects on Schwann cell proliferation and synaptic formation and contribute to the repair of PNS injury [78]. With the progress of electrospinning nanofiber membrane technology and neural tissue engineering-related research, it is believed that injurious diseases of the peripheral nervous system will be better treated.

### Application of electrospun nanofiber membranes in skin tissue engineering

The skin is the largest organ in the body of vertebrates, and its main function is to provide a natural barrier between the body and the external environment. In addition, the skin has immune regulation, temperature regulation, absorption of external substances, secretion, excretion, metabolism, and other important functions [86].

Skin wounds occur when the structural integrity of the skin is damaged by a variety of factors. These factors may be tissue rupture due to trauma, burns, congenital abnormalities, diseases that cause physical or psychological distress, or even chronic defects. When a wound occurs, the structural integrity of the skin needs to be repaired in order not to interfere with homeostasis, inflammation, cell migration, proliferation, and maturation [87]. Though the skin has a strong self-healing ability and scar tissue on the skin will gradually recover after trauma or skin injury, in severe cases of injury scar tissue produces higher collagen during the healing process. This in turn causes the tissue to become harder than its natural part, and the new skin from this scar tissue is less elastic than the original skin. Thus, the application of skin tissue engineering is needed in such an instance.

Skin tissue engineering-derived skin substitutes for wound healing have been widely used in clinical practice and have been developed extensively in recent years, with many new materials being carefully evaluated and awaiting approval for clinical use [88]. Electrospun materials can simulate the ECM of skin tissue very accurately and are among the ideal materials for skin tissue engineering.

Electrospun nanofiber membrane materials for skin tissue engineering have developed rapidly in recent years. Many researchers have conducted experimental studies to find suitable materials for skin tissue engineering. Fathi developed co-electrospun composites of polyvinyl alcohol, chitosan, and silk mat; the results of the experimental analysis suggested that the new fibrous structure could be used in the repair of damaged skin and regenerative medical applications as a skin substitute [89] (Fig. 4b). Sirsendu et al. used co-blended electrospinning to obtain a silk-gelatin loaded hybrid cationic gelatin/hyaluronic acid/chondroitin sulfate nanofiber composite scaffold, which was experimentally shown to promote adhesion and proliferation of hMSCs to a certain extent and to be effective for skin tissue repair [90]. Arturo et al. applied a novel recombinant biomaterial called the system for obtaining fibers from elastin-like recombinants. It can be cross-linked directly to the formed nanofibers during the flight of the fiber jet from the needle tip to the collection electrode during the spinning process, either as a dressing to promote skin cell growth or it can be directly implanted into the injured area as a tissue-engineered skin substitute [91].

## Application of electrospun nanofiber membranes in wound dressing

During wound healing of the skin, the electrospinning membrane is not only used as a substitute for skin tissue engineering; it is also widely used as a wound dressing to promote wound healing. Skin is a very fragile and easily damaged organ; therefore, there has been a lot of research interest in the repair of skin wounds. The normal process of wound healing consists of four stages: hemostasis, inflammation, proliferation, and ECM remodeling. The healing process is primarily regulated by interactions between various types of cells, bioactive factors, and supporting platforms (usually natural ECM secreted by cells). Under pathophysiological conditions, the healing process of the body will be severely disrupted. For example, severe injuries caused by burns or accidents cause a loss of skin tissue that exceeds the body's normal ability to heal, leading to an inability to heal. Most hard-to-heal wounds are related to large wounds from accidents or diseases such as diabetes. These conditions, which often require surgical treatment such as skin grafts, have become a major medical burden over the years [92].

An ideal wound dressing should absorb excess wound exudate while keeping the wound moist. This effectively protects the wound from microorganisms, allowing gas exchange and facilitating wound healing [93, 94]. In addition, the dressing material should be non-toxic, non-allergenic, easy to apply or remove, and have suitable adhesion. Traditional wound dressings, such as gauze, bandages, and sponges, are used to prevent bacterial invasion, but their limited ability to expand limits their use [95]. Modern wound dressings, such as hydrogels and nanofibers generated by electrospinning naturally sourced active ingredients, have been extensively explored to overcome this problem in regenerative medicine and trauma [96].

**Fig. 5:**
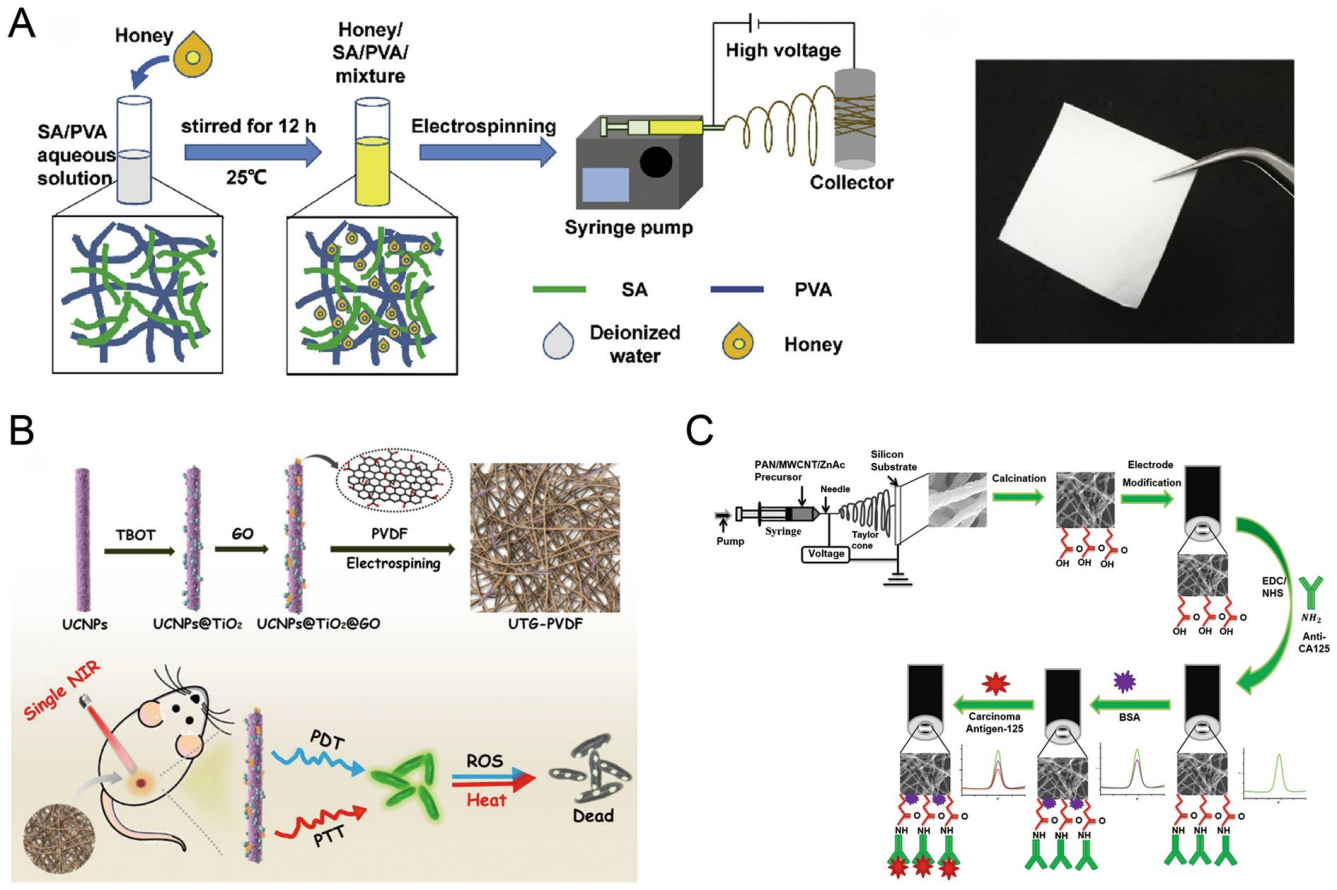
**a** The production process and the photograph of honey/SA/PVA nanofiber membrane [97]. Reprint with permission from Elsevier; **b** Synthesis process of UTG-PVDF nanocomposite film, and its sterilization principle under near-infrared light irradiation [98]. Reprint with permission from American Chemical Society; **c** Construction of carcinoma antigen-125 immunosensor and schematic diagram of action principle [104]. Reprint with permission from Elsevier

In recent years, the electrospinning nanofiber membrane materials used as a wound dressing to promote wound healing have emerged one after another. Traditional healing drugs have been used to load fibrous membrane scaffolds. For example, a study using honey mixed with alginate/polyvinyl alcohol-based electrospun nanofiber membranes showed good antioxidant and antibacterial activity, which is beneficial for wound healing [97] (Fig. 5a). In other studies, photosensitizer (PS) materials were used in electrospinning to obtain nanocomposite films. As a key component of photodynamic therapy, PS materials can produce cytotoxic reactive oxygen species by visible or ultraviolet light irradiation, and they show significant antibacterial properties. The results showed that the nanofiber membrane could effectively inhibit inflammation and control infection in wound healing, showing its application potential as wound dressing [98] (Fig. 5b). In addition, it is reported that Shi et al. constructed self-pumping dressings as bio-fluid pumps by combining the fiber film electrospun from the hydrophobic polyurethane nanofiber array with a hydrophilic microfiber network such as medical gauze. This self-pumping dressing can unidirectionally drain excess biofluid from the hydrophobic side to the hydrophilic side of the wound, thus preventing these biofluids from wetting the wound and causing infection, and greatly accelerating the healing process [73]. Yang et al. used mechanical growth factor to surface modify electrospun PCL fiber scaffolds. This modification directed macrophage phenotypic conversion, effectively reducing wound foreign body reaction in healing [99]. Electrospinning nanofiber membranes have strong plasticity and can be easily fabricated with different properties after changing the materials, conditions, and methods of electrospinning. As such, their popularity in the field of wound dressings remains high.

## Application of electrospun nanofiber membranes in cancer diagnosis and treatment

Cancer is a disease caused by mutated cells, whose ability to proliferate indefinitely and escape apoptosis eventually leads to the formation of tumors and subsequent invasion of surrounding tissues [100]. Currently, the most common treatment for cancer is the use of chemotherapy drugs. According to the World Health Organization, a total of 9.6 million people died of cancer in 2018, making it the second leading cause of death globally, with the majority of cases occurring in low- and middle-income countries [101]. Early detection and effective treatment have always been key factors in improving the survival rate of cancer patients. In addition to the increased incidence of cancer, cancer-related morbidity and mortality are further exacerbated by the development of tumor resistance to some existing chemotherapeutic drugs. As the disease progresses, the need for new therapies increases; however, the high cost of developing new drugs, high failure rates, and long trial cycles mean that new therapies are urgently needed for cancer treatment [101].

### Application of electrospun nanofiber membranes for immunosensor/cancer screening

The early detection and treatment of cancer are crucial for the improvement of survival rates. However, early cancers are usually asymptomatic and hard to detect. The characteristic biomarkers of cancer tumor overexpression are likely to appear in the early stage of the disease, and their identification and detection may be the best way to identify early malignant tumors. This has led to the emergence of a variety of cancer biomarker-sensing technologies [102].

The latest advances in nanoscience and nanotechnology have opened up new fields for the development of biosensors. Electrospun nanofilament membrane materials are comparable in size to biomolecules and offer high sensitivity and selectivity while providing device miniaturization and unaffected performance [102]. The use of nanofibers in areas such as volatile analysis, tumor imaging, and fluid sample analysis has accelerated the early diagnosis of cancer and helped improve patient survival [103].

Recent research on nanofiltration membrane sensors related to early tumor diagnosis has not been successful. Brince et al. synthesized highly oriented zinc oxide (ZnO) nanowires embedded in multi-walled carbon nanotubes using electrospinning and the fabrication of a novel biosensor platform based on multi-walled carbon nanotubes embedded in zinc oxide nanowires for the ultra-sensitive detection of tumor antigen125. This antigen is the tumor marker of ovarian cancer and the gold standard of diagnosis [104] (Fig. 5c). More sensors included in clinical research and application will promote the early diagnosis and treatment of cancer, thereby improving greatly the survival rate of cancer patients.

### Application of electrospun nanofiber membranes for drug delivery in the treatment of cancer

Nanofiber electrospinning is becoming the preferred process for the development of a variety of novel drug delivery systems (DDSs), owing to its simplicity, cost-effectiveness, and unique top-down preparation process [105]. The high flexibility of electrospun nanofibers enhances the control of drug release kinetics, simultaneously delivers different therapeutic drugs, and promotes local therapeutic effects, thereby reflecting the advantages of electrospun nanofibers in drug delivery system applications [106].

As drug delivery systems, electrospun nanofiber scaffolds are easy to operate, have low toxicity and good therapeutic effects, and are often used as targeted drug delivery tools [100]. This is a promising anticancer drug delivery technology, especially in postoperative topical chemotherapy, which allows for direct topical drug delivery to the tumor site and precise temporal and spatial control of the release of therapeutic drugs [100]. Balan et al. studied the anticancer effect of drug-loaded nanofibers with A431 cells by preparing PCL nanofibers loaded with chitosan nanoparticles. When the nanoparticle and nanofiber scaffold treatments were administered, the survival rate of A431 cells decreased by 30% and 50%, respectively. This indicates that the nanofiber delivery system opens a new frontier in the field of cancer therapy with reduced side effects and efficient cancer targeting [107]. Kita et al. investigated the ability of an electrospun polylactic acid drug reservoir device (DRD) to prolong the delivery time of the ear protectant metformin and established an in vitro model using SH-SY5Y human neuroblastoma cells. These results suggest that electrospinning DRD could provide a drug delivery system for customizable chemotherapeutic drugs that could have broad clinical applications in the personalized delivery of inner ear treatment [108]. Implantable biopolymer DDSs will be more widely used in clinical practice as more polymers are discovered for the construction of electrospun nanofiber delivery systems. In addition to cancer therapy, electrospun nanofibrous scaffolds are widely used as drug delivery systems for wound dressing, growth factor delivery, nucleic acid delivery, and stem cell delivery [109].

## Application of electrospun nanofiber membranes in medical protective equipment

The COVID-19 pandemic has caused shortages in medical protective clothing equipment. This has put many people, especially health care workers, at high risk [110]. There is an increasing demand for effective antimicrobial and antiviral protective devices that are easy to use.

The electrospun nanofiber membrane material is favored in the manufacturing field of medical protective equipment such as masks and protective clothing because of its good surface porosity and chemical functionalized surface that greatly enhances the filtration performance of nanomembranes. Moreover, it also has good air permeability and is comfortable.

**Fig. 6:**
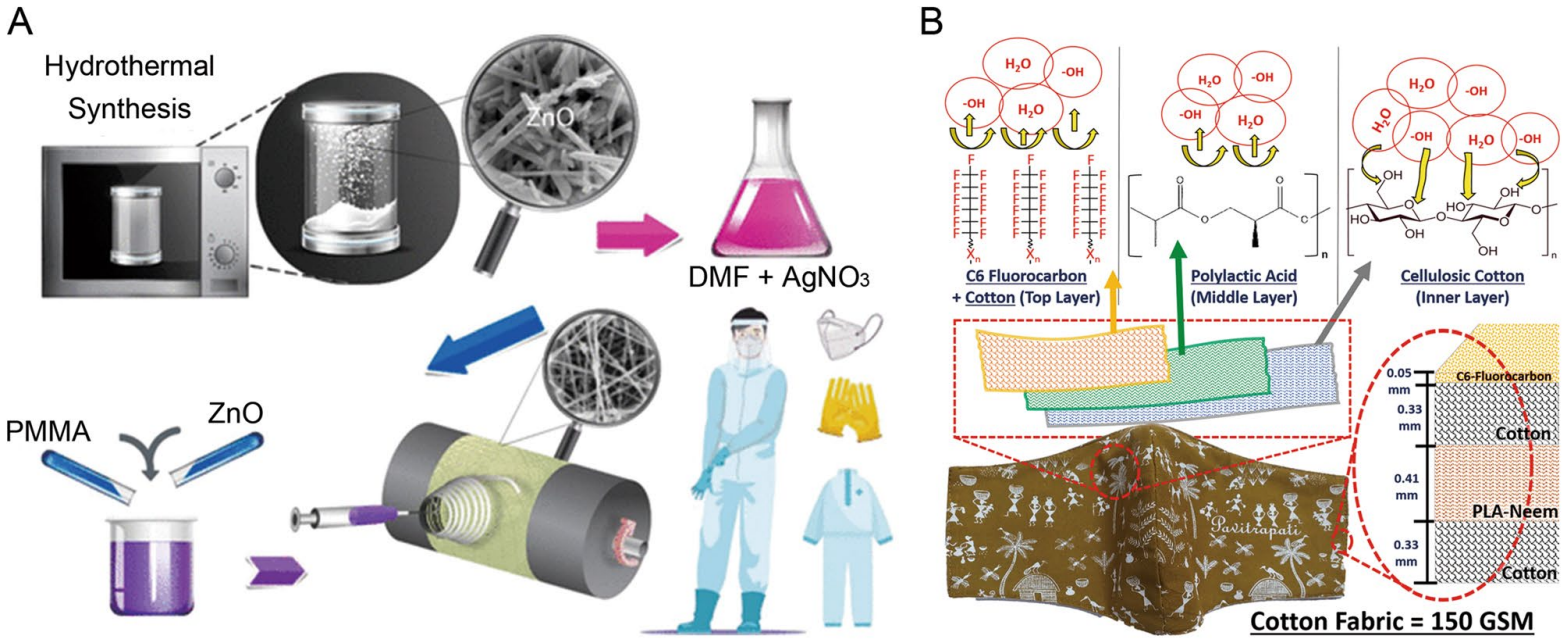
**a** PMMA/ZnO−Ag Nanoparticles (Ag NFS) was prepared by electrospinning method with this solution, which was used to make protective clothing [111]. Reprint with permission from American Chemical Society; **b** Preparation of polylactic acid nanofiber filter layer by needle-less electrospinning is used to manufacture three-layer cotton-PLA-cotton layered biodegradable mask [112]. Reprint with permission from Elsevier

In the past 2 years, the scientific community has seen an explosive increase in the research of electrospun nanofiber membranes in medical protective devices. A multifunctional electrospinning polymethyl methacrylate (PMMA) nanofiber with ZnO nanorods and silver (Ag) nanoparticles (PMMA/ZnO−Ag Nanoparticles) as decorative materials for making protective cushions have been reported. This material has antibacterial and antiviral properties, facilitates the photocatalytic degradation of organic pollutants, and can be reused [111] (Fig. 6a). Patil et al. reported a layered biodegradable mask with a polylactic acid nanofiber filter layer prepared using needleless electrospinning, which is effective in pandemic prevention [112] (Fig. 6b). Shao et al. developed a janus fiber membrane material that provides comprehensive protection with a focus on both dust and directional water transfer capabilities [113]. Another study reported that the a protective material made of superhydrophobic microporous membrane that is both waterproof and breathable can be made via electrospinning [114]. Additionally, it has high potential in the application of protective clothing. These facts demonstrate that the nanofiber membrane materials produced by electrospinning play an important role in a variety of fields.

## Conclusions and future perspectives

Compared with nanofibrous membrane materials obtained using other techniques, nanofibrous membrane materials obtained using electrospinning have unique advantages (high plasticity, specific surface area, and porosity). Obtaining nanofibrous membranous scaffolds using electrospinning technology is simpler and cheaper than most other technologies. Its industrial mass production is being explored by large instrument companies, and various new electrospinning technologies are being developed (there is also continuous development of various novel electrospinning technologies). The ease of nanosurface modification and the versatility of spinning material options have allowed researchers to explore new scaffold constructions, with breakthroughs in bone and cartilage, vascular grafts, artificial heart valves, and bionic myocardial tissue. The small diameter and adjustable pore size of electrostatically spun nanofiber films have also shown advantages in the manufacture of wound dressings and medical protective gear. Solvent-free or green solvent electrospinning methods have also received much attention in recent years, especially in the medical field. Electrospinning with green solvents to obtain nanofiber structures capable of replacing nanofiber components with chemical components can effectively avoid a range of side effects [115] and can effectively enhance the effectiveness of drugs [116]. It is evident that the application of electrospun nanofiber film materials should continue to develop and grow in the foreseeable future.

Although electrostatically spun nanofiber membranes have facilitated significant advances in tissue engineering, cancer therapy, and drug delivery, some problems remain to be resolved. For example, the electrospinning process is not applicable to certain electrically sensitive materials, especially biomolecules, resulting in a relatively narrow range of applications. In addition, it requires operation under high voltage conditions, which may pose a safety risk to the operator. In addition, electrospinning technology is mostly used in laboratory research; large-scale industrial production of the technology is still not very mature. However, given that a large number of researchers are continuing to explore and develop multi-needle technology, needle-free technology, and AC electrospinning technology, demonstrating their potential for large-scale industrial production, this problem should gradually be solved. Finally, based on the simplicity, feasibility, and cost-effectiveness of electrospinning technology and its broad application prospect in the biomedical field, it is expected to develop rapidly in the coming years.
